# Topological magnetic structure generation using VAE-GAN hybrid model and discriminator-driven latent sampling

**DOI:** 10.1038/s41598-023-47866-3

**Published:** 2023-11-21

**Authors:** S. M. Park, H. G. Yoon, D. B. Lee, J. W. Choi, H. Y. Kwon, C. Won

**Affiliations:** 1 Department of Physics, Kyung Hee University, Seoul, 02447 South Korea; 2 Department of Battery-Smart Factory, Korea University, Seoul, 02841 South Korea; 3 Center for Spintronics, Korea Institute of Science and Technology, Seoul, 02792 South Korea

**Keywords:** Computational science, Magnetic properties and materials

## Abstract

Recently, deep generative models using machine intelligence are widely utilized to investigate scientific systems by generating scientific data. In this study, we experiment with a hybrid model of a variational autoencoder (VAE) and a generative adversarial network (GAN) to generate a variety of plausible two-dimensional magnetic topological structure data. Due to the topological properties in the system, numerous and diverse metastable magnetic structures exist, and energy and topological barriers separate them. Thus, generating a variety of plausible spin structures avoiding those barrier states is a challenging problem. The VAE-GAN hybrid model can present an effective approach to this problem because it brings the advantages of both VAE’s diversity and GAN’s fidelity. It allows one to perform various applications including searching a desired sample from a variety of valid samples. Additionally, we perform a discriminator-driven latent sampling (DDLS) using our hybrid model to improve the quality of generated samples. We confirm that DDLS generates various plausible data with large coverage, following the topological rules of the target system.

## Introduction

Driven by the successful advances of deep generative models in the past few years, scientific data generation has become one of the essential topics in the scientific research field. Novel computational approaches based on deep generative models have been developed to generate scientific data from experimental or simulation datasets. The deep generative models stand for deep neural networks which are designed to generate synthetic data by approximating complicated and high-dimensional data distribution^1^. The two representative deep generative models, variational autoencoder (VAE)^2^ and generative adversarial network (GAN)^3^, are extensively used for scientific research and data generation. For example, VAEs have been used for new physics mining at the Large Hadron Collider^4^ and molecular designing^5^, whereas GANs have been used for weather prediction^6^ and CT image augmentation^7^.

In condensed matter physics, the magnetic system is a representative system where deep learning techniques are actively applied^8–10^. This is not only because unique physical characteristics appear due to the competition of several complicated magnetic interactions, but also because several toy models based on magnetic systems (such as the Ising and Heisenberg models) are generally used to analyze the physical phenomena observed in various research fields. Among the deep learning techniques, VAE-based models have been widely adapted to various magnetic systems for investigating phase transition behaviors^11–15^, characterizing crystal structures^16^, interpolating and extrapolating magnetic structures^17^, searching for optimal magnetic structures^18^, estimating effective fields^19^, and finding the ground states^20, 21^.

Despite these successful applications, the sampling data for topological spin structures using VAE still has certain limitations, which originated from the representative disadvantage of VAE called the latent space smoothness^22^. In the usual training process of VAE, the latent space is formed to follow a simple prior distribution (e.g., Gaussian distribution). It means that complicated target data distributions may not be accurately represented by the simple continuous latent space of VAE, and it leads to difficulties in capturing intricate relationships between the data points. Also in the case of sampling data for topological spin structures, each of the spin structures is distinctly separated with high energy barriers induced by topological properties, but VAE cannot learn the details of topological difference between the spin structures. Consequently, it allows the generation of non-plausible spin structures with topological defects including nodal points^18, 21^. The previous studies address this issue using prior scientific knowledge of the target system, such as adding the energy of generated spin configurations to the cost function^21^ or polishing the generated samples to lower their energies^18^. However, prior knowledge is often absent for other datasets, thus additional studies are needed for generating topological data avoiding topological defects without any prior knowledge.

On the other hand, the various deep generative models based on GANs are also applied to generate scientifically plausible data in various research fields of condensed matter physics^23, 24^. In a usual GAN model, a discriminator network learns to distinguish between real and fake samples while a generator network is trained to produce samples that are realistic enough to deceive the discriminator network. Because of the adversarial relationship between the discriminator and generator, the GAN-based models have shown greater capabilities in photo-realistic data generation compared with the VAE^25^. Analogously, it is expected that the GAN-based models can produce more realistic spin structures with no or fewer nodal points. However, a problem of GAN-based models is that the diversity of generated samples is poorer than VAE-based models^26^. To secure both the high plausibility of GAN-based models and the high diversity of VAE-based models simultaneously, recently the hybrid models of VAE and GAN are intensively studied and applied to various research fields^27–29^.

The goal of this study is to implement a generator which can produce physically and topologically reliable magnetic structures. To achieve our goal, we build a hybrid model of two representative deep generative models, VAE and GAN, and train it with two-dimensional spin structures to exhibit its strengths as a topological structure generator. The results are compared with those of standalone VAE and GAN. The trained models are quantitatively evaluated by coverage and energy metrics that measure the diversity and fidelity of generated samples, respectively. We visualize the latent space manifolds of the trained models to analyze the underlying reasons for topological defects that appeared in generated samples. Additionally, we suggest that the discriminator-driven latent sampling (DDLS) method^30–32^ can be applied to improve the plausibility of generated samples by eliminating topological defects.

## Strategy

### VAE-GAN hybrid model

**Figure 1:**
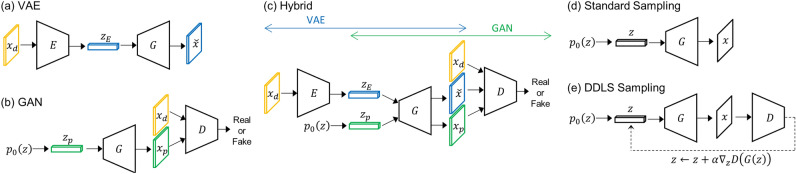
(**a**–**c**) Illustration of the training workflows of (**a**) VAE, (**b**) GAN, and (**c**) hybrid model. The $E$*,* $G$, and $D$ denote the encoder, generator (or decoder), and discriminator, respectively. (**d**) The schematic description of standard sampling in VAE, GAN, and hybrid model. (**e**) The schematic description of DDLS sampling. The dashed arrow indicates the gradient backpropagation and the $\alpha$ denotes the gradient step size.

The hybrid model combines the training workflows of VAE and GAN. For the comparative investigation, we train three models: VAE, GAN, and the hybrid model. The VAE is built with two neural networks of encoder $E$ and generator (decoder) $G$ as shown in Fig. 1a. The encoder converts training data ${x}_{d}$ into a latent code ${z}_{E}$, and the generator decodes ${z}_{E}$ to $\widetilde{x}$. On the other hands, the GAN consists of generator $G$ and discriminator $D$ as shown in Fig. 1b. A random latent code ${z}_{p}$ is sampled from a prior distribution ${p}_{0}(z)$ and becomes a fake data ${x}_{p}$ via the generator. Then, the real data ${x}_{d}$ and fake data ${x}_{p}$ are classified by the discriminator. The hybrid model has three neural networks, encoder, generator, and discriminator. Figure 1c illustrates the overall training workflow of the hybrid model. Note that the reconstructed data $\widetilde{x}$ is also fed into the discriminator as fake data. Therefore, unlike a standalone GAN, the number of fake samples is twice the number of real samples.

The loss function of the hybrid model also contains both components of VAE and GAN losses. The loss function of VAE, ${\mathcal{L}}^{\mathrm{VAE}}$, is shown in Eq. (1), where $N$ is the dimensionality of input features ($128\times 128\times 3)$, $\beta$ is a coefficient of the regularization term^33^, and ${p}_{E}\left(z|x\right)$ is the posterior distribution of the encoder. The terms ${\mathbb{E}}$ and ${D}_{\mathrm{KL}}$ denote expectation value and the Kullback–Leibler divergence, respectively.

1 $${\mathcal{L}}^{{{\text{VAE}}}} = \frac{1}{N}{\mathbb{E}}\left[ {\left( {x_{d} - \tilde{x}} \right)^{2} } \right] + \beta D_{KL} \left( {p_{E} \left( {z{|}x} \right)p_{0} \left( z \right)} \right).$$

The first term measures how well the VAE can reconstruct the input data and the second term enforces the trained latent space of the VAE to be close to the prior distribution, ${p}_{0}\left(z\right)$, which is the Gaussian distribution in this study.

For the GAN loss functions, we use the non-saturating losses^3^ as shown in Eq. (2), where ${\mathcal{L}}_{D}^{\mathrm{GAN}}$ and ${\mathcal{L}}_{G}^{\mathrm{GAN}}$ denote the loss functions for the discriminator and generator, respectively.

2 $$\begin{gathered} {\mathcal{L}}_{D}^{{{\text{GAN}}}} = - {\mathbb{E}}\left[ {\log \left( {D\left( {x_{d} } \right)} \right)} \right] - {\mathbb{E}}\left[ {\log \left( {1 - D\left( {x_{p} } \right)} \right)} \right], \hfill \\ {\mathcal{L}}_{G}^{{{\text{GAN}}}} = - {\mathbb{E}}\left[ {\log \left( {D\left( {x_{p} } \right)} \right)} \right]. \hfill \\ \end{gathered}$$

The discriminator learns to classify real and fake data with ${\mathcal{L}}_{D}^{\mathrm{GAN}}$, the loss function of discriminator. The first term, $-{\mathbb{E}}\left[\mathrm{log}\left(D\left({x}_{d}\right)\right)\right]$, represents the expectation of the negative log probability that the discriminator assigns to real data, denoted by ${x}_{d}$, being real. The second term, $-{\mathbb{E}}\left[\mathrm{log}\left(1-D\left({x}_{p}\right)\right)\right]$, represents the expectation of the negative log probability that the discriminator assigns to data produced by the generator, denoted by ${x}_{p}$, being fake. It means that the discriminator learns to classify the real and fake data. On the other hand, the loss function of generator, $-{\mathbb{E}}\left[\mathrm{log}\left(D\left({x}_{p}\right)\right)\right]$, represents the negative log probability that the discriminator assigns to the fake data ${x}_{p}$ being real.

Finally, the entire loss functions of the hybrid model are shown in Eq. (3), where $\gamma$ is the coefficient of the GAN loss.

3 $$\begin{gathered} {\mathcal{L}}_{E}^{{{\text{Hybrid}}}} = {\mathcal{L}}^{{{\text{VAE}}}} , \hfill \\ {\mathcal{L}}_{D}^{{{\text{Hybrid}}}} = - {\mathbb{E}}\left[ {\log \left( {D\left( {x_{d} } \right)} \right)} \right] - \frac{1}{2}{\mathbb{E}}\left[ {\log \left( {1 - D\left( {x_{p} } \right)} \right)} \right] - \frac{1}{2}{\mathbb{E}}\left[ {\log \left( {1 - D\left( {\tilde{x}} \right)} \right){ }} \right], \hfill \\ {\mathcal{L}}_{G}^{{{\text{Hybrid}}}} = {\mathcal{L}}^{{{\text{VAE}}}} + \gamma \left( { - \frac{1}{2}{\mathbb{E}}\left[ {\log \left( {D\left( {x_{p} } \right)} \right)} \right] - \frac{1}{2}{\mathbb{E}}\left[ {\log \left( {D\left( {\tilde{x}} \right)} \right)} \right]} \right). \hfill \\ \end{gathered}$$

These loss functions are separately used to train the encoder, discriminator, and generator. Detailed training conditions and hyperparameters are described in the Experimental Section.

### Discriminator-driven latent sampling

The standard sampling method of deep generative models, including VAE and GAN, is sampling a random latent code $z$ from a prior distribution (e.g., the standard normal distribution) and feeding it into the generator network, as shown in Fig. 1d. In GAN-based models, there is another approach to generate new samples, called discriminator-driven latent sampling (DDLS). At the end of training GAN, the adversarial game between the generator and discriminator generally does not converge to the generator’s ground truth (generating extremely realistic data and completely deceiving the discriminator), thus the discriminator still watches for the implausibility of generated data. Based on this fact, previous studies have proposed various DDLS methods such as rejecting unrealistic samples^34^ and polishing implausible factors of generated samples using a Monte Carlo method^30–32^ via the discriminator evaluation.

We implement a simple DDLS algorithm, as shown in Fig. 1e, and use it to improve the topological plausibility of generated samples. In the algorithm, a latent code is initially sampled from a prior distribution, and it is iteratively updated using a gradient descent method to maximize the evaluation of the trained discriminator, $D\left(G\left(z\right)\right)$. Since the discriminator is trained to return a value of one for real data and zero for fake data, the process of maximizing the discriminator’s evaluation is expected to evolve the generated spin configuration, $G\left(z\right)$, to become more realistic (without topological defects). The results of DDLS are presented later in the Results Section.

### Dataset

We train the VAE, GAN, and hybrid models on two-dimensional metastable spin structures which have various labyrinth patterns^35^. The dataset is generated by a simulated annealing process implemented by the Monte Carlo method. We suppose the Heisenberg model with the Hamiltonian $\mathcal{H}=-J{\sum }_{\langle i,j\rangle }{\overrightarrow{S}}_{i}\cdot {\overrightarrow{S}}_{j}-{\sum }_{\langle i,j\rangle }{\overrightarrow{D}}_{ij}\cdot \left({\overrightarrow{S}}_{i}\times {\overrightarrow{S}}_{j}\right)$ in a square lattice of $128 \times 128$ grid sites with a periodic boundary condition, where $J$, ${\overrightarrow{D}}_{ij}$, and ${\overrightarrow{S}}_{i}$ denote the exchange interaction parameter, the Dzyaloshinskii-Moriya interaction vector^36, 37^, and the Heisenberg spin on the $i$-th grid site, respectively. The parameters $J$ and $\left|{\overrightarrow{D}}_{ij}\right|$ are fixed at 1.0 and 0.3, respectively. In this system, the spin configurations are determined by the spontaneous symmetry-breaking process, so that we can generate countless different metastable states under the fixed condition. We generate a total of 40,000 spin configurations, which are divided into 30,000 training datasets and 10,000 test datasets.

### Metrics

Our goal is to evaluate the diversity and fidelity of each data generation strategy based on the VAE, GAN, and hybrid model with and without the DDLS. Unfortunately, many representative metrics, such as the Inception Score^38^ or Fréchet Inception distance^39^, evaluate generative models without discerning between diversity and fidelity. Furthermore, these metrics are properly available only to the models trained on the ImageNet dataset^40^ because they need a reference model pre-trained on the same dataset to embed the generated samples into the feature space of the reference model. For these reasons, we measure the diversity using the coverage metric^26^ which counts the mass of real data “covered” by the model distribution implied in a generator network. To evaluate the fidelity of generated spin configurations, we simply measure the energy using the Hamiltonian $\mathcal{H}$ because the spin configurations in our dataset are the results of the simulated annealing process so that they are energetically stabilized.

## Results

### Comparison between the VAE, GAN, and hybrid models

**Figure 2:**
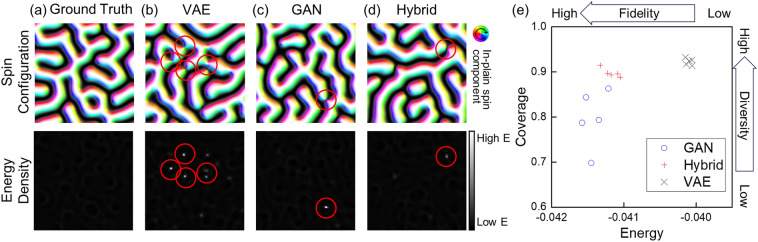
(**a**) A sample spin configuration in our test dataset and its energy density map. (**b**–**d**) Spin configurations generated by the trained (**b**) VAE, (c) GAN, and (**d**) hybrid models, and their energy density maps. The colors and black/white contrast of the spin configurations indicate the in-plane and out-of-plane directions of local spins, respectively. Red circles highlight the positions of nodal points. (**e**) Coverage and energy metric values of the trained models. Each scatter point indicates the result of independent trials.

The hybrid model shown in Fig. 1a is trained with our dataset and losses discussed in the Strategy Section, and the standalone VAE and GAN are also trained under the same conditions to be compared with the hybrid model. Figure 2a–d show the comparison between the ground truth (a sample of spin configuration in our test dataset) and spin configurations generated from each of the trained models. The spin configuration generated by the trained VAE model exhibits lots of nodal points, as indicated by the red circles. In contrast, the spin configurations generated by the trained GAN and hybrid models show a smaller number of nodal points in comparison with that of the VAE model. These nodal points are implausible structures; they are the topological defects in this magnetic system, thus they do not appear in the ground truth spin configuration. In addition, these nodal points are energetically unstable; in the energy density maps, there are high-energy peaks (bright points) at the positions of the nodal points exist. It means that we can measure the plausibility or fidelity of the generated spin configurations by their energy, and the number of nodal points is strongly related to them. By these facts, one can check that the fidelity of the GAN and hybrid models surpasses that of the VAE model.

To quantitatively investigate the coverage and energy metric values, we independently perform the training processes five times and generate 10,000 samples for each trained model as shown in Fig. 2e. Higher coverage values indicate better diversity and lower energy values indicate better plausibility; the upper left part of the graph in Fig. 2e indicates better results. As mentioned above, we confirm that both GAN and hybrid models produce lower energy samples compared with the VAE. This is consistent with the conventional understanding that usual GAN-based generative models have the advantage to generate more realistic samples than VAE-based models. On the other hand, the coverages of the VAE and hybrid models are around 0.92 and 0.90 on average, respectively, whereas the coverage of GAN is only 0.80 on average. Consequently, we confirm that the hybrid model takes advantages of both the high coverage of VAE and the high fidelity of GAN simultaneously. Another advantage of the hybrid model is that it is more stable than the standalone GAN. This can be observed from the fact that the coverage and energy metric values of the five independent trials of hybrid models show less dispersion compared to those from the standalone GANs.

### Latent space analysis

**Figure 3:**
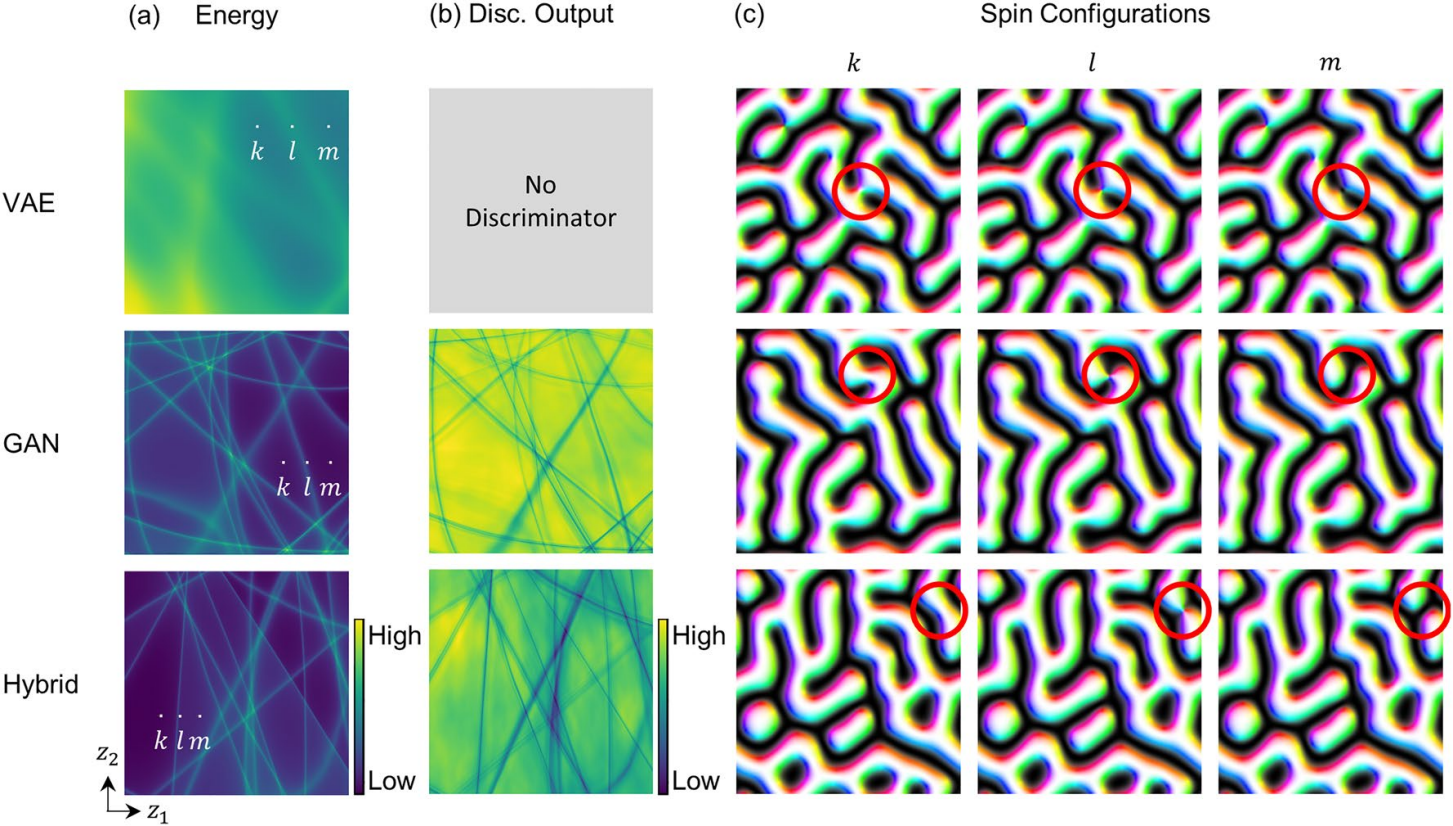
Latent space visualization. (**a**–**b**) Heatmaps for the representations of (**a**) energy values and (b) discriminator logits along two axes, ${z}_{1}$ and ${z}_{2}$, within a range from − 2 to 2. The brighter(darker) color in (**a**) and (**b**) indicates the higher(lower) energy values and the real-like(fake-like) samples in the perspective of the discriminators, respectively, for each trained model (no discriminator in VAE). (**c**) Spin configurations decoded from the latent codes on the $k$, $l$, and $m$ positions in (a). Red circles are highlighting the changing structures.

To investigate the underlying characteristics of the deep generative models trained with the topological magnetic structure dataset, we analyze the latent space manifolds of the models as shown in Fig. 3. To display the high-dimensional latent space of each model, we arbitrarily select two axes, ${z}_{1}$ and ${z}_{2}$, out of a total $n$ axes ($n=128$, where $n$ is the dimensionality of a latent space) and sample various latent codes by scanning a specific region of the chosen two-dimensional latent space. The other $n-2$ components of latent codes are fixed as the random numbers sampled from the standard normal distribution which is the prior distribution of our models. It is verified that the choice of axes does not make any significant differences in the discussion of this section. The sampled latent codes are decoded into the spin configurations through the trained decoder or generators of VAE, GAN, and hybrid models for calculating the energy and discriminator logits of them. To plot the calculated values on the chosen two-dimensional latent space, we use heatmap representations as shown in Fig. 3a for the energies and (b) for the discriminator logits on the same latent space region.

The most interesting feature in Fig. 3a is that, compared with the blurred energy heatmap of the VAE, the energy heatmaps of the GAN and hybrid models include several narrow lines and flat regions surrounded by the lines. This interesting feature is also shown in the heatmaps for the discriminator logits of the GAN and hybrid models in Fig. 3b. Considering that the spin configurations in our dataset are distinctly separated by topological properties with high energy barriers, the latent spaces partitioned into several flat regions by the narrow lines may imply that the GAN and hybrid models properly learn the topological properties in our dataset. Specifically, the narrow lines are supposed to be indicating the specific regions in the latent spaces which can be decoded into high energy states with emerging implausible topological defects, implying the boundaries between the energetically stabilized but topologically different spin configurations.

Figure 3c directly supports that the narrow lines are closely related to the emergence of implausible topological defects. For each of the VAE, GAN, and hybrid models, three spin configurations are decoded from the latent codes at three different latent positions, $k$, $l$, and $m$ marked in Fig. 3a. Within each latent space, $k$ and $m$ represent two separate flat regions and $l$ is on the boundary line between them. As indicated by the red circles shown in Fig. 3c, the spin configurations from $k$ and $m$ exhibit topologically distinct local spin structures, while the spin configuration from $l$ includes a nodal point.

Consequently, we confirm that the latent spaces formed by the training with our topological data are composed of multiple latent domains (flat regions) and latent domain walls (boundaries between the latent domains), which are strongly related to the topological properties implied in the dataset. In addition, it is also confirmed that the latent domains and latent domain walls are clearly distinguished in the latent spaces of the GAN and hybrid models, whereas the latent space of VAE is blurred overall than the other models.

### Data generation using DDLS

As discussed in the previous section, the GAN and hybrid models considered in this study have an advantage in generating plausible data by narrowing down the latent regions which can be decoded into implausible data. However, there is still a possibility that a latent code is sampled from the narrow regions. To generate new samples without implausible defects, we apply the DDLS algorithm shown in Fig. 1e using the generator and discriminator in a trained hybrid model.

**Figure 4:**
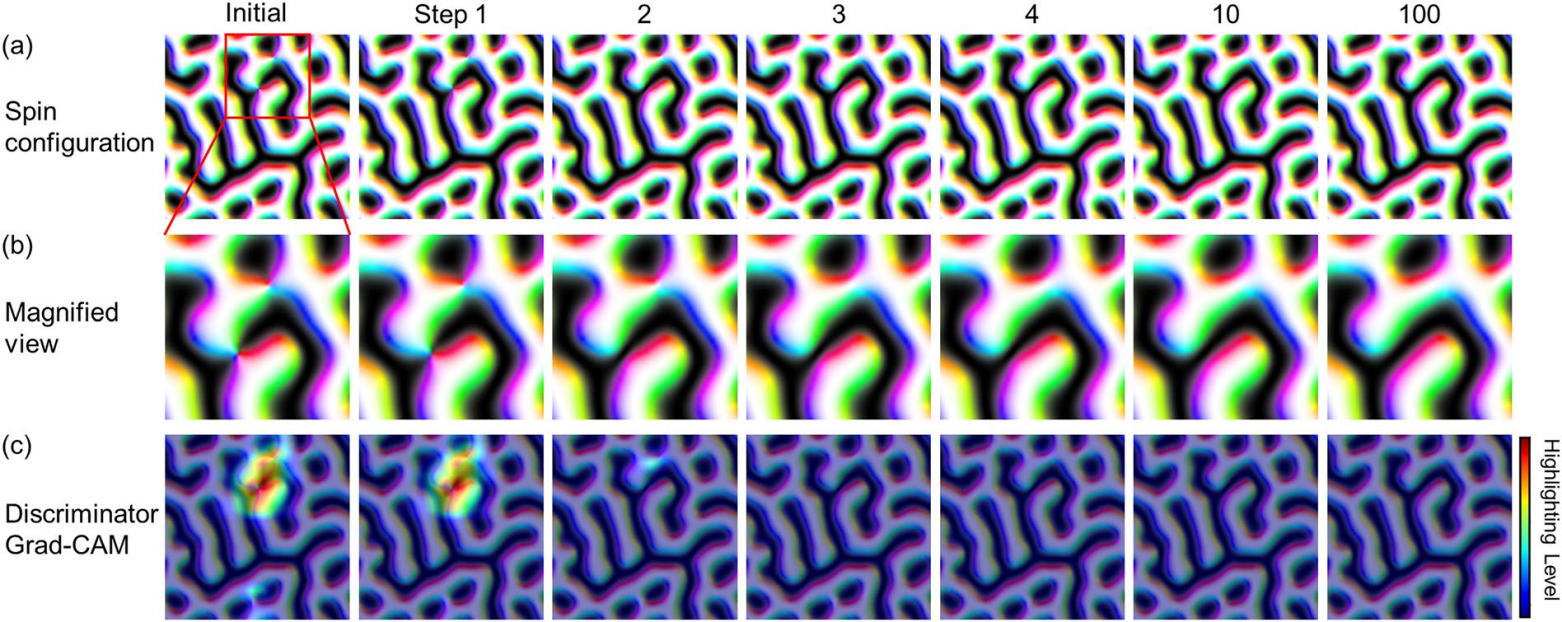
Data generation process using the DDLS algorithm. (**a**) Changes in spin configuration during the iterative process of the DDLS algorithm. The red square in the initial spin configuration highlights a nodal point region. (**b**) Magnified view of the highlighted region in (**a**). (**c**) Grad-CAM of each spin configuration. The red color indicates an important area for the discriminator to predict the spin configuration as fake.

Figures 4a and b show the results of the DDLS algorithm. As the DDLS progresses, the initial spin configuration becomes energetically stabilized by removing several nodal points; for example, the nodal points within the local spin structures highlighted by the red box in the initial spin configuration are removed after the first few iterations. After 100 iteration steps, the initial spin configuration evolves into a new spin configuration without any implausible topological defects.

To offer an insight into how the DDLS algorithm can eliminate the nodal points in the initial spin configurations, we plot a gradient-based class activation map (Grad-CAM)^41^ as shown in Fig. 4c to demonstrate the role of the discriminator in the DDLS algorithm. The Grad-CAM is an explanation method for convolutional neural network classifiers including the discriminators in the GAN and hybrid models. It shows the specific regions in the input data that significantly influence the decision of classifiers. Obviously, the Grad-CAM result on the initial spin configuration highlights the position of nodal points, indicating the nodal points are crucial factors for the discriminator to determine the spin configuration as a fake data. As the nodal points gradually disappear during the DDLS progresses, the level of highlighting is reduced. This means that it becomes more challenging for the discriminator to determine the spin configuration as a fake data.

Consequently, it is confirmed that the crucial factor for the discriminator to classify a spin configuration as a real or fake data is the existence of nodal points, which are the implausible topological defects in our target system. Using the discriminator, we can utilize the DDLS algorithm to generate topologically plausible data by removing implausible defects as shown in Fig. 4.

### Applications of the hybrid Model

The trained hybrid model is not only capable of generating plausible data but is also applicable for various other purposes. In this section, we demonstrate two application examples. The first example is searching for the optimal solutions for various objectives, including maximizing out-of-plane magnetization ${M}_{z}$, minimizing ${M}_{z}$, and minimizing energy $\epsilon$ of a spin configuration. Utilizing the well-trained generator in our hybrid model, various optimization algorithms can be implemented within the latent space^18^. Specifically, we can search for the optimal solutions according to defined objectives by obtaining the corresponding latent codes. We employ a genetic algorithm, a conventional optimization algorithm inspired by the biological evolution processes^42^. The second example is investigating intermediate states between the optimal spin configurations. It also can be performed in the latent space of the hybrid model by interpolating between the latent codes corresponding to the optimal solutions.

**Figure 5:**
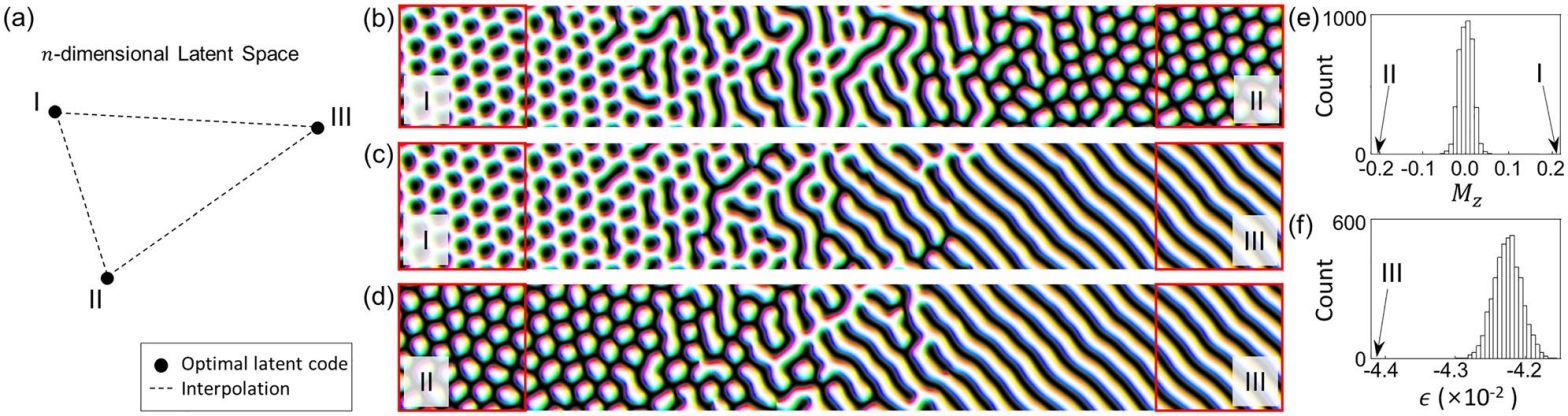
Application of the trained hybrid model. (**a**) A schematic illustration of interpolation in the latent space. Three dots, I, II, and III, represent the optimized latent codes obtained by maximizing ${M}_{z}$, minimizing ${M}_{z}$, and minimizing $\epsilon$, respectively. (**b**–**d**) Spin configurations obtained by the linear interpolation between (**b**) I and II, (**c**) I and III, and (**d**) II and III. The red squares indicate each optimal spin configuration. (**e**) The ${M}_{z}$ distribution of test dataset. The ${M}_{z}$ values of the optimal solutions I and II are indicated by arrows. (**f**) The $\epsilon$ distribution of test dataset and the $\epsilon$ value of the optimal solution III.

Figure 5a schematically illustrates the locations of optimized latent codes and the interpolation lines, where the I, II, and III represent the latent codes obtained by maximizing ${M}_{z}$, minimizing ${M}_{z}$, and minimizing $\epsilon$, respectively. The results of optimizing ${M}_{z}$ (either by maximizing or minimizing) are skyrmion lattices, highlighted within the red squares on the left and right sides of Fig. 5b. Minimizing $\epsilon$ results in well-aligned stripe structures, highlighted within the red squares on the right sides of Fig. 5c, d. The ${M}_{z}$ values of the optimal solutions are 0.211 and − 0.204, respectively, which represent extreme values compared to the ${M}_{z}$ distribution of the training dataset, which has mean of 0.000 with a standard deviation of ± 0.016 (see Fig. 5e). The optimized $\epsilon$ value is − 0.0442, which is significantly lower than that of the training dataset: $\epsilon$ has a mean of − 0.0423 with a standard deviation of ± 0.0002 (see Fig. 5f). These optimal solutions, the skyrmion lattices and well-aligned stripe structure, are physically reliable. In a magnetic system, the ${M}_{z}$ can be controlled by applying an out-of-plain external field. Numerous studies reported that the labyrinth structures become skyrmion structures when the external field is applied^43, 44^. The well-aligned stripe structures are also observed as the ground state of the system in previous studies^35, 45^.

The optimization process using our trained model is completed in a short period of time (In our study, it takes approximately 20 min), whereas it is impossible to achieve the goal in a reasonable time frame with conventional micromagnetic simulations. Notably, the ground state of the system, characterized by a well-aligned stripe structure, remains elusive to conventional micromagnetic simulations due to the existence of numerous metastable states. The computational efficiency of optimization using our hybrid model is attributed to the dimensionality reduction achieved through the trained generative model. While the original system has dimensions of 128 × 128 × 3, the latent space of our model is 128-dimensional, which substantially narrows the target space where we search for optimal solutions. In the realm of optimization, higher dimensions dramatically increase the complexity of the problem and constrain the efficiency of algorithms applied. Our hybrid model effectively reduces this dimensionality, which facilitates the successful application of the genetic algorithm within the dimensionally reduced latent space of the model. We believe this optimization strategy can be applied to a wide range of systems and objectives, such as designing molecules or materials. The approach is also adaptable to other conventional optimization algorithms, including gradient-based methods and Monte Carlo methods.

It is important to highlight that, as we mentioned above, the optimized solutions generated by the hybrid model are absent from the training dataset. At a same time, they are physically reliable, not displaying any node points. This indicates that the hybrid model is capable of generating new, physically reliable samples, extending beyond the training dataset. We confirm that the hybrid model can generate a wide variety of samples (high diversity) with high fidelity.

The central areas in Fig. 5b–d illustrate the results of linear interpolation within the latent space: from the latent code I to II (b), from I to III (c), and from II to III (d). The interpolated structures transition smoothly from one optimal state to another, exhibiting few node points and maintaining physically appropriate configurations under varying out-of-plane magnetization ${M}_{z}$ and energy $\epsilon$. The reliability of these interpolated structures can be ascribed to the adversarial training, contrasting with the increased number of node points observed in interpolations performed within the latent space of a standalone VAE model. We confirm that our hybrid model possesses advantages, including the capability to generate a diverse of new, physically reliable samples and the potential for application across various domains.

## Conclusion

We investigate the performance of a VAE-GAN hybrid model as a generator for the data with topological properties. We generate a dataset composed of various spin configurations which are simulated on a two-dimensional magnetic system and use it to train a simple VAE-GAN hybrid model. The performance of the trained hybrid model is evaluated in the aspects of diversity and fidelity, and it is compared with those of standalone VAE and GAN models. It is confirmed that the hybrid model exhibits high diversity and high fidelity simultaneously by incorporating the strengths of both standalone VAE and GAN models. Through the latent space visualization, we find that the latent space built by each model is partitioned into the numerous latent domains by latent domain walls which are closely related to the topological properties implied in our dataset. We show that, even if a topologically implausible structure appears on a generated sample, the DDLS algorithm can improve the plausibility of the sample by removing the appearing topological defects. Finally, we demonstrate two application examples of the trained hybrid model, which are searching for the optimal solutions for various objectives and investigating intermediate states between them. We believe that the hybrid model has great potential as a generator for topological data, offering numerous versatile applications.

## Experimental section

### Neural network structures

**Figure 6:**
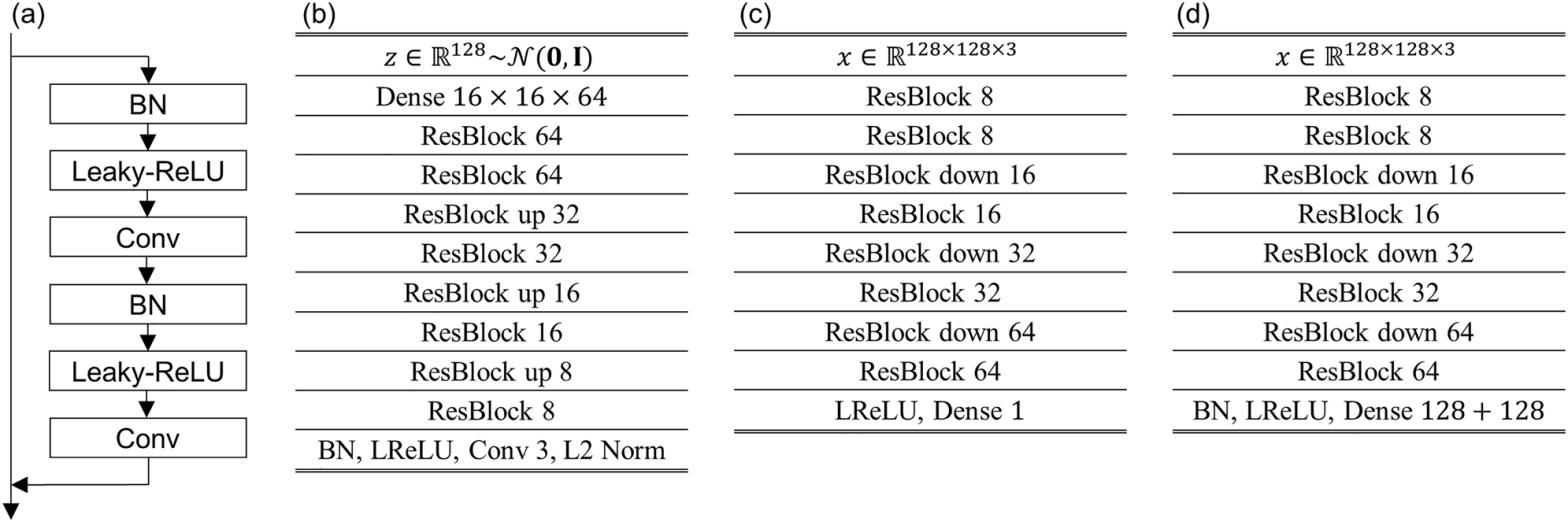
Neural networks used in this study. (**a**) A schematic illustration of the pre-activation residual block. For the discriminator, the batch normalization layers are removed. (**b**–**d**) The network architecture of the (**b**) generator, (**c**) discriminator, and (**d**) encoder. The BN and LReLU denote batch normalization and Leaky-ReLU activation, respectively.

For the neural networks, the pre-activation residual building blocks (ResBlocks)^46, 47^ with batch-normalization and Leaky-ReLU activation function are used, as shown in Fig. 6a. Figure 6b–d shows the generator, discriminator, and encoder architectures. Periodic padding is applied before all convolutions because of the periodic boundary condition of the dataset. The spectral normalization^48^ is applied for the discriminator layers, thus the batch normalization layers were removed from the discriminator network. To force the generator output pixels to be the Heisenberg spins (normalized pixels), the pixels were normalized at the end of the generator. The Up sampling of $2\times 2$ is applied for upsizing in the generator, whereas the downsizing in the discriminator and encoder is implemented by a convolutional layer with a stride size of 2 and kernel size of $4\times 4$.

### Hyperparameters

The $\beta$, coefficient of the regularization loss term in the VAE loss, is chosen by $0.001$. The $\gamma$, coefficient of the GAN loss component in the generator of the hybrid model, is chosen by 0.001. For training, we use Adam optimizers^49^ with the learning rate, ${\beta }_{1}$, and ${\beta }_{2}$ of 0.0002, 0.0, and 0.9, respectively. The training dataset contains 30,000 samples. The batch size is 50 and the number of total training steps is 6000 (100 epochs).

## Data Availability

The dataset used in this work is available at https://data.mendeley.com/datasets/4833bhfjv3/1.

## References

[1] Ruthotto L, Haber E (2021). An introduction to deep generative modeling. GAMM Mitteilungen.

[2] Kingma, D. P., Welling, M. Auto-encoding variational bayes. In *2nd International Conference on Learning Representations, ICLR 2014—Conference Track Proceedings* (2014).

[3] Goodfellow IJ, Pouget-Abadie J, Mirza M, Xu B, Warde-Farley D, Ozair S (2014). Generative adversarial nets. Adv. Neural Inf. Process. Syst..

[4] Cerri O, Nguyen TQ, Pierini M, Spiropulu M, Vlimant JR (2019). Variational autoencoders for new physics mining at the Large Hadron Collider. J. High Energy Phys..

[5] Lee YJ, Kahng H, Kim SB (2021). Generative adversarial networks for de novo molecular design. Mol. Inform..

[6] Bihlo A (2021). A generative adversarial network approach to (ensemble) weather prediction. Neural Netw..

[7] Sandfort V, Yan K, Pickhardt PJ, Summers RM (2019). Data augmentation using generative adversarial networks (cycleGAn) to improve generalizability in CT segmentation tasks. Sci. Rep..

[8] Kwon HY, Yoon HG, Lee C, Chen G, Liu K, Schmid AK (2020). Magnetic Hamiltonian parameter estimation using deep learning techniques. Sci. Adv..

[9] Kwon HY, Kim NJ, Lee CK, Won C (2019). Searching magnetic states using an unsupervised machine learning algorithm with the Heisenberg model. Phys. Rev. B.

[10] Chen X, Araujo FA, Riou M, Torrejon J, Ravelosona D, Kang W (2022). Forecasting the outcome of spintronic experiments with Neural Ordinary Differential Equations. Nat. Commun..

[11] Wetzel SJ (2017). Unsupervised learning of phase transitions: From principal component analysis to variational autoencoders. Phys. Rev. E.

[12] Hu W, Singh RRP, Scalettar RT (2017). Discovering phases, phase transitions, and crossovers through unsupervised machine learning: A critical examination. Phys. Rev. E.

[13] Acevedo S, Arlego M, Lamas CA (2021). Phase diagram study of a two-dimensional frustrated antiferromagnet via unsupervised machine learning. Phys. Rev. B.

[14] Vlcek L, Ziatdinov M, Maksov A, Tselev A, Baddorf AP, Kalinin SV (2019). Learning from imperfections: Predicting structure and thermodynamics from atomic imaging of fluctuations. ACS Nano.

[15] Elias DR, Granato E, de Koning M (2022). Global exploration of phase behavior in frustrated Ising models using unsupervised learning techniques. Phys. A Stat. Mech. Appl..

[16] Routh PK, Liu Y, Marcella N, Kozinsky B, Frenkel AI (2021). Latent representation learning for structural characterization of catalysts. J. Phys. Chem. Lett..

[17] Yoon HG, Lee C, Lee DB, Park SM, Choi JW, Kwon HY (2022). Interpolation and extrapolation between the magnetic chiral states using autoencoder. Comput. Phys. Commun..

[18] Park SM, Yoon HG, Lee DB, Choi JW, Kwon HY, Won C (2022). Optimization of physical quantities in the autoencoder latent space. Sci. Rep..

[19] Lee DB, Yoon HG, Park SM, Choi JW, Kwon HY, Won C (2021). Estimating the effective fields of spin configurations using a deep learning technique. Sci. Rep..

[20] Kwon HY, Yoon HG, Park SM, Lee DB, Shi D, Wu YZ (2022). Searching for the ground state of complex spin-ice systems using deep learning techniques. Sci. Rep..

[21] Kwon HY, Yoon HG, Park SM, Lee DB, Choi JW, Won C (2021). Magnetic state generation using Hamiltonian guided variational autoencoder with spin structure stabilization. Adv. Sci..

[22] Christopher. Understanding disentangling in β-VAE. *Osteologie* **25** (2016).

[23] Nouira, A., Sokolovska, N. & Crivello, J. C. CrystalGAN: Learning to discover crystallographic structures with generative adversarial networks. In *CEUR Workshop Proc* Vol. 2350 (2019).

[24] Kim S, Noh J, Gu GH, Aspuru-Guzik A, Jung Y (2020). Generative adversarial networks for crystal structure prediction. ACS Cent. Sci..

[25] Sami, M. & Mobin, I. A comparative study on variational autoencoders and generative adversarial networks (2019).

[26] Tolstikhin, I., Gelly, S., Bousquet, O., Simon-Gabriel, C. J. & Schölkopf, B. AdaGAN: Boosting generative models. *Adv. Neural Inf. Process. Syst.* (2017).

[27] Yu, X., Zhang, X., Cao, Y. & Xia, M. Vaegan: A collaborative filtering framework based on adversarial variational autoencoders. *In IJCAI International Joint Conference on Artificial Intelligence* Vol. 2019. 10.24963/ijcai.2019/584 (2019).

[28] Xian, Y., Sharma, S., Schiele, B. & Akata, Z. F-VAEGAN-D2: A feature generating framework for any-shot learning. In *Proceedings of the IEEE Computer Society Conference on Computer Vision and Pattern Recognition* Vol. 2019. 10.1109/CVPR.2019.01052 (2019).

[29] Cheng M, Fang F, Navon IM, Zheng J, Tang X, Zhu J (2022). Spatio-temporal hourly and daily ozone forecasting in china using a hybrid machine learning model: Autoencoder and generative adversarial networks. J. Adv. Model. Earth Syst..

[30] Che, T., Zhang, R., Sohl-Dickstein, J., Larochelle, H., Paull, L., Cao, Y. *et al.* Your GAN is secretly an energy-based model and you should use discriminator driven latent sampling. *Adv. Neural Inf. Process. Syst.* (2020).

[31] Tanaka, A. Discriminator optimal transport. *Adv. Neural Inf. Process. Syst.* **32** (2019).

[32] Turner, R., Hung, J., Frank, E., Saatci, Y. & Yosinski, J. Metropolis-Hastings generative adversarial networks. In *36th International Conference on Machine Learning, ICML 2019* Vol. 2019 (2019).

[33] Higgins, I., Matthey, L., Pal, A., Burgess, C., Glorot, X., Botvinick, M. *et al.* Β-VAE: Learning basic visual concepts with a constrained variational framework. In *5th International Conference on Learning Representations, ICLR 2017—Conference Track Proceedings* (2017).

[34] Azadi, S., Odena, A., Olsson, C., Darrell, T. & Goodfellow, I. Discriminator rejection sampling. In *7th International Conference on Learning Representations, ICLR, 2019* (2019).

[35] Kwon HY, Bu KM, Wu YZ, Won C (2012). Effect of anisotropy and dipole interaction on long-range order magnetic structures generated by Dzyaloshinskii-Moriya interaction. J. Magn. Magn. Mater..

[36] Moriya T (1960). New mechanism of anisotropic superexchange interaction. Phys. Rev. Lett..

[37] Dzyaloshinsky I (1958). A thermodynamic theory of “weak” ferromagnetism of antiferromagnetics. J. Phys. Chem. Solids.

[38] Salimans, T., Goodfellow, I., Zaremba, W., Cheung, V., Radford, A. & Chen, X. Improved techniques for training GANs. *Adv. Neural Inf. Process. Syst.* (2016).

[39] Heusel, M., Ramsauer, H., Unterthiner, T., Nessler, B. & Hochreiter S. GANs trained by a two time-scale update rule converge to a local Nash equilibrium. *Adv. Neural Inf. Process. Syst.* (2017).

[40] Deng, J., Dong, W., Socher, R., Li, L. J., Kai. L. & Li, F. F. ImageNet: A large-scale hierarchical image database. 10.1109/cvpr.2009.5206848 (2010).

[41] Selvaraju RR, Cogswell M, Das A, Vedantam R, Parikh D, Batra D (2020). Grad-CAM: Visual explanations from deep networks via gradient-based localization. Int. J. Comput. Vis..

[42] Goldberg DE, Holland JH (1988). genetic algorithms and machine learning. Mach. Learn..

[43] Yu XZ, Onose Y, Kanazawa N, Park JH, Han JH, Matsui Y (2010). Letters Real-space observation of a two-dimensional skyrmion crystal. Nature.

[44] Bogdanov A, Hubert A (1994). Thermodynamically stable magnetic vortex states in magnetic crystals. J. Magn. Magn. Mater..

[45] Uchida M, Onose Y, Matsui Y, Tokura Y (1979). Real-space observation of helical spin order. Science.

[46] He, K., Zhang, X., Ren, S. & Sun, J. Deep residual learning for image recognition. *In Proceedings of the IEEE Computer Society Conference on Computer Vision and Pattern Recognition* Vol. 2016. 10.1109/CVPR.2016.90 (2016).

[47] He, K., Zhang, X., Ren, S. & Sun, J. Identity mappings in deep residual networks. In *Lecture Notes in Computer Science (including subseries Lecture Notes in Artificial Intelligence and Lecture Notes in Bioinformatics)* Vol 9908 (LNCS, 2016). 10.1007/978-3-319-46493-0_38.

[48] Miyato, T., Kataoka, T., Koyama, M. & Yoshida, Y. Spectral normalization for generative adversarial networks. In *6th International Conference on Learning Representations, ICLR 2018—Conference Track Proceedings* (2018).

[49] Kingma, D. P. & Ba, J. L. Adam: A method for stochastic optimization. In *3rd International Conference on Learning Representations, ICLR 2015—Conference Track Proceedings* (2015).

